# Supplementary figures and images for: Dynamic proton-dependent motors power type IX secretion and gliding motility in Flavobacterium
Source: PLoS Biol. 2022 Mar 25;20(3):e3001443. doi: 10.1371/journal.pbio.3001443 (PMC8986121; doi:10.1371/journal.pbio.3001443)

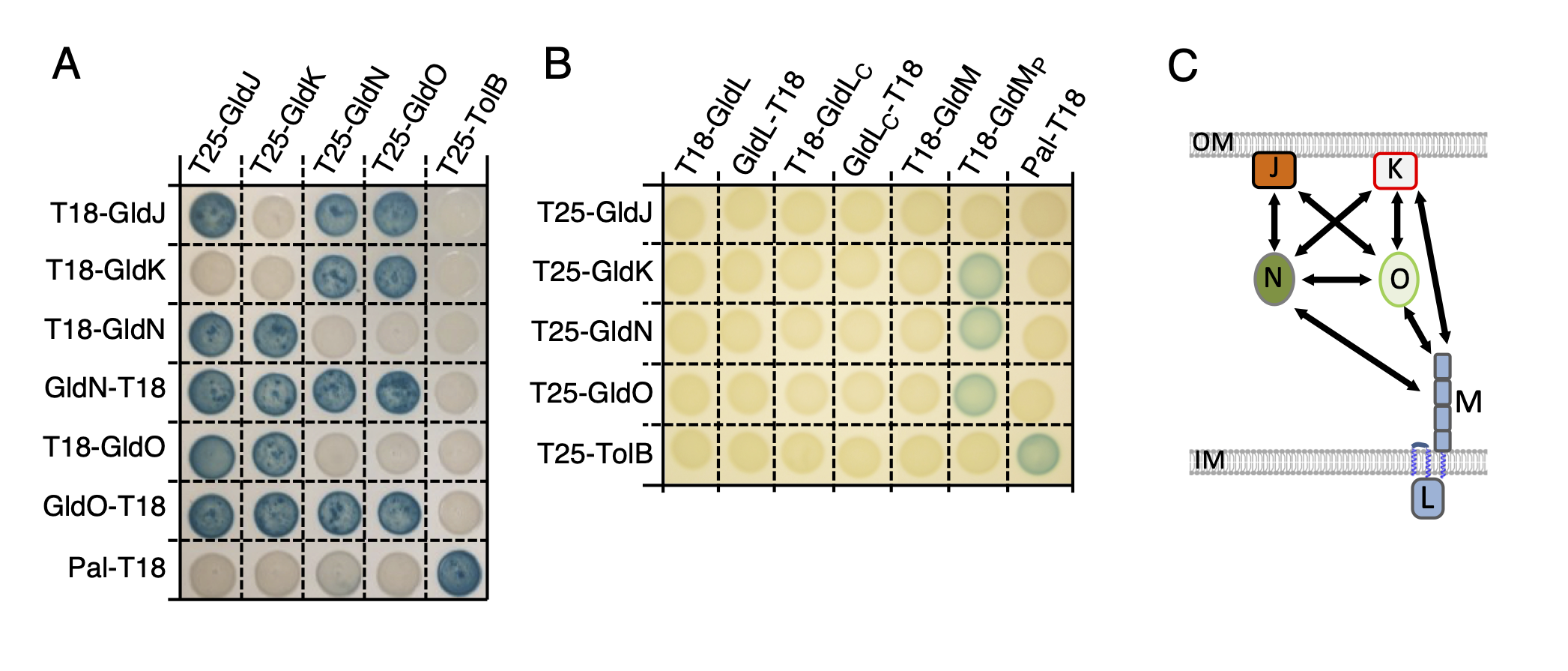

Supplement: S1 Fig — Bacterial 2-hybrid assays. (A) T9SS OM-associated core complex (GldK, GldN and GldO) and GldJ. (B) T9SS OM-associated core complex (GldK, GldN, and GldO), GldJ and the IM-associated core complex (GldM and GldL). The signal sequence was omitted in the constructs for GldN and GldO. The signal sequence and the acylated N-terminal cysteine residue of the mature form were omitted for GldK and GldJ. BTH101 reporter cells producing the indicated proteins or domains (GldLC, cytoplasmic domain of the GldL protein; GldMP, periplasmic domain of the GldM protein) fused to the T18 or T25 domain of the Bordetella adenylate cyclase were spotted on plates supplemented with IPTG and the chromogenic substrate X-Gal. The TolB-Pal interaction serves as positive control. (C) Model of the interactions between T9SS components defined by bacterial 2-hybrid assay. IM, inner membrane; IPTG, iso-propyl-β-D-thio-galactopyranoside; OM, outer membrane; T9SS, type IX secretion system. (TIFF) [file pbio.3001443.s001.tiff]

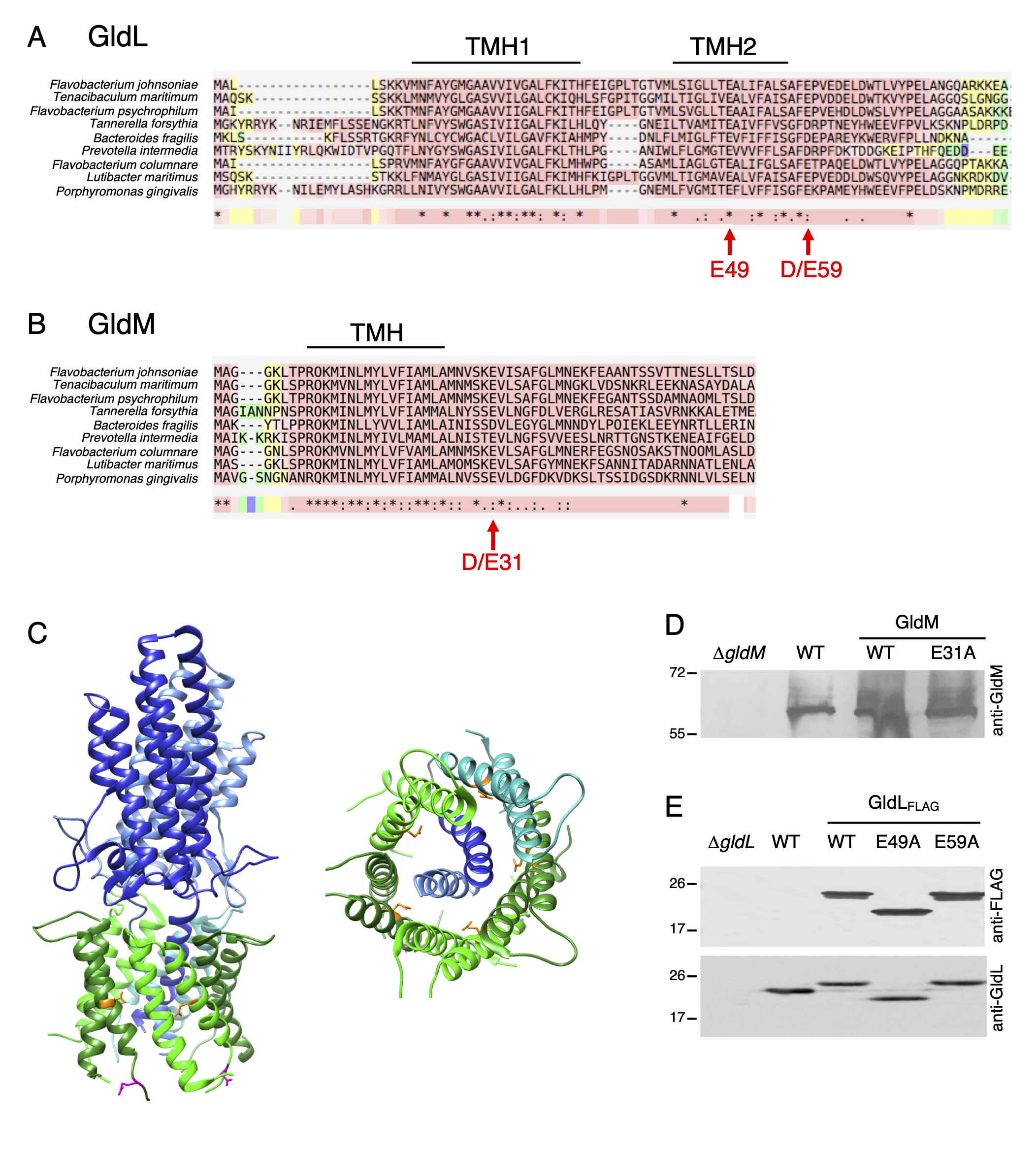

Supplement: S2 Fig — (A) Sequence alignments of the N-terminal regions that encompass the 2 transmembrane segments of GldL homologs. The alignment was performed using TCOFFEE. Red arrows indicate the conserved acidic residues. (B) Sequence alignments of the region that encompasses the single transmembrane segment of GldM homologs. The alignment was performed using TCOFFEE. The red arrow indicates the conserved acidic residue. The TMH regions (as defined by James and colleagues [42]) are indicated above the alignments. (C) Highlight of GldL-E49 (orange) and E59 (pink) glutamate residues in the structural model of the GldLM complex. The left panel shows a side view and the right panel shows a view from the cytoplasm. GldL TMHs are colored green. GldM subunits (TMH and first periplasmic domain) are colored blue. (D) Western blot analysis of GldM production using anti-GldM antibodies in a ΔgldM mutant, WT F. johnsoniae, GldM WT or GldM E31A expressed from a plasmid in a ΔgldM mutant background. (E) Western blot analysis of GldL production using anti-GldL antibodies in the ΔgldL mutant (ΔgldL), WT F. johnsoniae, and strains expressing GldLWT-flag (GldLWT) or GldLE49A-flag (E49A) or GldLE59A-flag (E59A). Extracts of cells were subjected to SDS-PAGE and immunodetection with anti-GldL and anti-Flag primary antibodies and HRP-coupled secondary antibodies. Molecular mass markers (in kDa) are indicated on left. TMHs, transmembrane helices; WT, wild-type. (TIFF) [file pbio.3001443.s002.tiff]

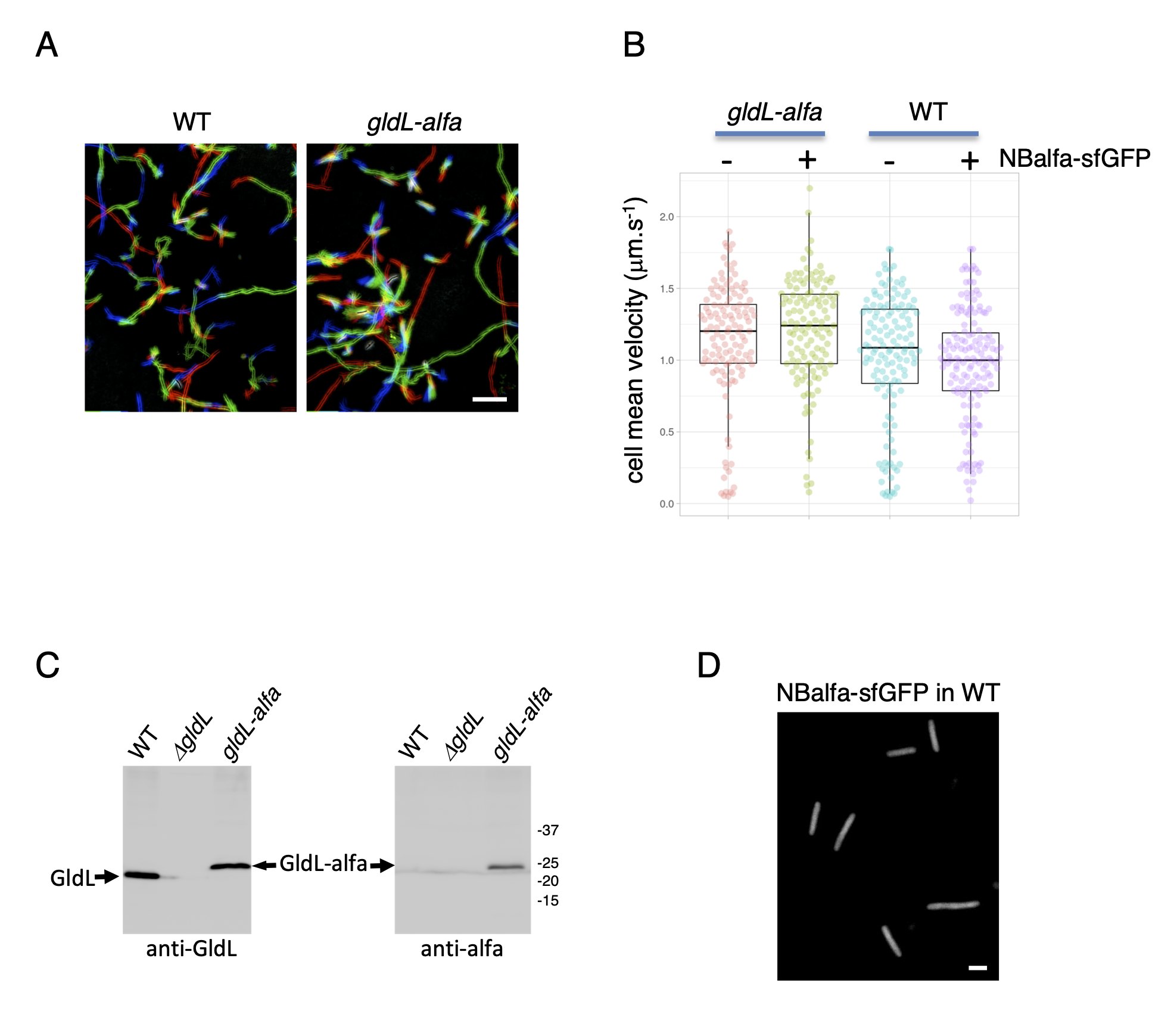

Supplement: S3 Fig — (A) Rainbow traces of cell motility on glass recorded by phase contrast microscopy over time (2 minutes) in a WT strain and a strain expressing gldL-alfa at the native locus. Individual frames from time-lapse acquisition were colored from red (start) to yellow, green, cyan, and blue (end) and merged into a single rainbow image. Scale bar, 20 μm. (B) Combined jitter plots/boxplots of mean cell gliding velocity (in μm.s−1) of gldL-alfa cells in the absence of NBalfa-sfGFP (−, n = 124) or with 1 mM IPTG induction of NBalfa-sfGFP for 1 hour (+, n = 125) or WT cells in the absence of NBalfa-sfGFP (−, n = 135) or with 1 mM IPTG induction of NBalfa-sfGFP for 1 hour (+, n = 151). Data underlying this figure can be found in S1 Data. (C) Western blot analysis of GldL-alfa production using anti-GldL polyclonal antibodies or anti-alfa NBalfa nanobodies in WT F. johnsoniae, in the ΔgldL mutant (ΔgldL) or in the strain expressing gldL-alfa at the native locus. Molecular mass markers (in kDa) are indicated on right. (D) Representative micrograph of cells expressing fluorescent NBalfa-sfGFP in a WT background. Scale bar, 2 μm. IPTG, iso-propyl-β-D-thio-galactopyranoside; WT, wild-type. (TIFF) [file pbio.3001443.s003.tiff]
