# Supplementary material for: Dynamic proton-dependent motors power type IX secretion and gliding motility in Flavobacterium
Source: PLoS Biol. 2022 Mar 25;20(3):e3001443. doi: 10.1371/journal.pbio.3001443 (PMC8986121; doi:10.1371/journal.pbio.3001443)
Supplement: S1 Table — (DOC) [file pbio.3001443.s004.doc]

**S1 Table. Strains, plasmids and oligonucleotides used in this study.**

**STRAINS**

**Strains Description and genotype Source or Reference**

*Escherichia coli* K12

DH5α F-, Δ(*argF*-l*ac*)U169, *phoA*, s*upE44*, Δ(*lacZ*)M15, *relA*, *endA*, *thi*, *hsdR*  Laboratory collection

BTH101 F-, *cya99, araD139, galE15, gakK16, rpsL, hsdR, mcrAB* (Karimova *et al*., 1998)

HB101 strain containing the pRK2013 plasmid for triparental conjugation Laboratory collection

*Flavobacterium johnsoniae*

UW101 Wild-type *F. johnsoniae* Laboratory collection

∆*gldL* Deletion of the *gldL* gene Shrivastava *et al*., 2013

*gldL^E49A^* *gldL* point mutant This study

*gldL^E59A^* *gldL* point mutant This study

∆*gldM* Deletion of the *gldM* gene This study

∆*gldM, gldL^WT^* *gldM* deletion mutant expressing GldM^WT^ from a replicative plasmid This study

∆*gldM, gldL^E31A^* *gldM* deletion mutant expressing GldM^E31A^ from a replicative plasmid This study

∆*gldK* Deletion of the *gldK* gene Shrivastava *et* *al*., 2013

∆*gldN* Deletion of the *gldN* gene Shrivastava *et al*., 2013

*gldL-alfa* GldL-alfa tag fusion expressed from the native locus This study

*gldL-alfa, NBalfa-sfGFP* GldL-alfa tag fusion at the native locus and NBalfa-sfGFP fusion expressed from an IPTG inducible promoter This study

**PLASMIDS**

**Plasmid Description and main characteristics Source or reference**

pCP23 *E. coli-F. johnsoniae* shuttle vector, Amp^R^-Tet^R^ McBride and Kempf, 1996

pRR51 suicide vector containing a wild type *rpsL* allele, Amp^R^-Em^R^ Rhodes *et al*., 2011

pAML-L1 pAML100 plasmid with GldL TMH1 (residues Val8-Thr29) inserted between *NheI* and *Bam*HI This study

pBML-L1 pBML148 plasmid with GldL TMH1 (residues Val8-Thr29) inserted between *Nhe*I and *Bam*HI This study

pAML-M pAML100 plasmid with GldM TMH (residues Leu15-Leu38) inserted between *Nhe*I and *Bam*HI This study

pBML-M pBML148 plasmid with GldM TMH (residues Leu15-Leu38) inserted between *Nhe*I and *Bam*HI This study

pAML-TssL1 pAML100 plasmid with TssL TMH1 (residues Met183-Ala207) inserted between *Nhe*I and *Bam*HI Laboratory collection

pBML-TssL1 pBML148 plasmid with TssL TMH1 (residues Met183-Ala207) inserted between *Nhe*I and *Bam*HI Laboratory collection

pNBla1-L2 pN-BLA1.2 plasmid with GLdL TMH2 (residues Val40-Val61) inserted between *Nhe*I and *Bam*HI This study

pCBla1-L2 pC-BLA1.2 plasmid with GLdL TMH2 (residues Val40-Val61) inserted between *Nhe*I and *Bam*HI This study

pCBla2-L1 pC-BLA2.0 plasmid with GLdL TMH1 (residues Val8-Thr29) inserted between *Nhe*I and *Bam*HI This study

pCBla2-M pC-BLA2.0 plasmid with GLdM TMH (residues Leu15-Leu38) inserted between *Nhe*I and *Bam*HI This study

pNBla1-TssM2 pN-BLA1.2 plasmid with TssM TMH2 (residues Ile44-Met63) inserted between *Nhe*I and *Bam*HI Laboratory collection

pCBla2-TssM2 pC-BLA1.2 plasmid with TssM TMH2 (residues Ile44-Met63) inserted between *Nhe*I and *Bam*HI Laboratory collection

pRR51-Δ*gldM* Construct used to generate Δ*gldM* deletion

pRR51-*gldL*^E49A^ Construct used to generate *gldL*^E49A^ point mutant This study

pRR51-*gldL*^E59A^ Construct used to generate *gldL*^E59A^ point mutant This study

pCP23-gldL-gldM^WT^ replicative plasmid for *gldL* and *gldM*^WT^ expression in *F. johnsoniae*  This study

pCP23-gldL-gldM^E31A^ replicative plasmid for *gldL* and *gldM*^E31A^ expression in *F. johnsoniae*  This study

pT18-GldJ GldJ mature form cloned downstream of T18 into pUT18C This study

pT18-GldK GldK mature form cloned downstream of T18 into pUT18C This study

pT18-GldN GldN mature form cloned downstream of T18 into pUT18C This study

pGldN-T18 GldN mature form cloned upstream of T18 into pUT18 This study

pT18-GldO GldO mature form cloned downstream of T18 into pUT18C This study

pGldO-T18 GldO mature form cloned upstream of T18 into pUT18 This study

pPal-T18 Pal mature form cloned upstream of T18 into pUT18 Battesti and Bouveret, 2008

pT18-GldM_P_ GldM periplasmic domain cloned downstream of T18 into pUT18C This study

pT18-GldM GldM cloned downstream of T18 into pUT18C This study

pGldL_C_-T18 GldL cytosolic domain cloned upstream of T18 into pUT18 This study

pT18-GldL_C_ GldL cytosolic domain cloned downstream of T18 into pUT18C This study

pGldL-T18 GldL cloned upstream of T18 into pUT18 This study

pT18-GldL GldL cloned downstream of T18 into pUT18C This study

pT18-Pal Pal mature form cloned downstream of T25 into pUT18C Battesti and Bouveret, 2008

pT25-GldJ GldJ mature form cloned downstream of T25 into pKT25 This study

pT25-GldK GldK mature form cloned downstream of T25 into pKT25 This study

pT25-GldN GldN mature form cloned downstream of T25 into pKT25 This study

pT25-GldO GldO mature form cloned downstream of T25 into pKT25 This study

pT25-TolB TolB mature form cloned downstream of T25 into pKT25 Battesti and Bouveret, 2008

pRR51-gldL-alfa Construct used to generate gldL-alfa fusion at the native locus This study

pCPlac-NbAlfa-sfGFP Replicative plasmid to express NBalfa-sfGFP from an IPTG inducible promoter in *F. johnsoniae* This study

**OLIGONUCLEOTIDES**

**Oligonucleotide Sequence (5' to 3')**

Plasmid construction ^a,b^

For GALLEX and BLA

(oligonucleotide primers were rehybridized and used for insertion into plasmids digested with *Nhe*I and *BamH*I)

GALLEX-Bla-GldL1-up CTAGCAAAAAAGTTATGAATTTCGCTTATGGTATGGGAGCGGCAGTGGTAATCGTTGGAGCTTTATTCAAAATTACTAG

GALLEX-BLa-GldL1-bot GATCCTAGTAATTTTGAATAAAGCTCCAACGATTACCACTGCCGCTCCCATACCATAAGCGAAATTCATAACTTTTTTG

GALLEX-BLa-GldL2-up CTAGCGTGATGCTTTCTATCGGTCTTTTGACTGAGGCATTAATCTTTGCGTTATCTGCTTTTGAACCAGTTAG

GALLEX-BLa-GldL2-bot GATCCTAACTGGTTCAAAAGCAGATAACGCAAAGATTAATGCCTCAGTCAAAAGACCGATAGAAAGCATCACG

GALLEX-BLa-GldM1-up CTAGCCTGATGTATCTGGTTTTCATCGCAATGTTAGCAATGAACGTATCAAAAGAAGTTATTTCTGCTTTTGGTTTGAG

GALLEX-BLa-GldM1-bot GATCCTCAAACCAAAAGCAGAAATAACTTCTTTTGATACGTTCATTGCTAACATTGCGATGAAAACCAGATACATCAGG

GALLEX-BLa-TssM2-up CTAGCATACTACTGCTGATATTGCTGAGTCTGGGGGTGCTCTTTGTCTGTTACCTGCCTGTGATGAAAAAAAG

GALLEX-BLa-TssM2-bot GATCCTTTTTTTCATCACAGGCAGGTAACAGACAAAGAGCACCCCCAGACTCAGCAATATCAGCAGTAGTATG

For Bacterial two-hybrid (by restriction/ligation):

5-BACTH-gldJ GAAGTCTAGATAGCAAAAAATCGAGTTCGAGTCACG

3-BACTH-gldJ GAAGGGTACCCCATTTCTTGGTGATTTTCTTCTTTCAGTTTTAGCAC

5-BATCH-gldK GAAGTCTAGATTGTGGTAAGTCAGGTGACAAAGGTGAATTAG

3-BACTH-gldK GAAGGGTACCCCTTTTCTTCCGCCTCCTGTTACTTGAGTTCC

5-BACTH-gldN GAAGGGATCCTAATGCAAAAACTCCTGCTCAGATCGGACT

3-BACTH-gldN GAAGGGTACCCCGTAATTCCACATGTCTTGTTCGAAGTTACGAATC

5-BACTH-gldO GAAGTCTAGATAATGCTAAAACGGCTGATCAAATAGGGCAC

3-BACTH-gldO GAAGGGTACCCCGTAATTCCACATATCTTCTTCGAAGTTGCGAATC

5-BACTH-gldM-FL GAAGTCTAGATGCAGGAGGAAAATTAACCCCTAGACAG

5-BACTH-gldM-TMS GAAGTCTAGATTTTGGTTTGATGAATGAAAAATTCGAAGCTGCAAATAC

3-BACTH-gldM GAAGGGTACCCCTTGTATTTCGTAAATTACCGGAGCAGTTCTTG

5-BACTH-gldL-FL GAAGTCTAGATGCATTATTAAGTAAAAAAGTTATGAATTTCGCTTATGGTATGG

5-BACTH-gldL GAAGTCTAGATGAACCAGTTGAGGATGAATTAGATTGGACTCTAG

3-BACTH-gldL1 GAAGGGTACCCCTCCTTTGTTACTCATTGCAGAAAGCATACCAC

3-BACTH-gldL2 GAAGGGTACCCCTTGCTCTTTTAACTTAGCAGCGTTCTCAGC

For strain construction:

F1-ΔgldM (for restriction) GCTAGGGATCCGGTTCCGGGTGGATCTTTTATTATGGG

R1-ΔgldM (for restriction) GCTAGGTCGACGGTTAATTTTCCTCCTGCCATTTTTGTCT

F2-ΔgldM (for restriction) GCTAGGTCGACACTACGTTCAATATCTTATAATGATACCACG

R2-ΔgldM (for restriction) GCTAGGCATGCGCACTTCCCGCGCATTAGG

Fw-GldL-A49E TCTTTTGACTgcaGCATTAATCTTTGC

(for quick change mutagenesis on pRR51-gldL^E49A-E59A^)

Rv-GldL-A49E CCGATAGAAAGCATCACTG

(for quick change mutagenesis on pRR51-gldL^E49A-E59A^)

Fw-GldL-A59E TCTGCTTTTGaaCCAGTTGAGG

(for quick change mutagenesis on pRR51-gldL^E49A-E59A^)

Rv-GldL-A59E TAACGCAAAGATTAATGCTG

(for quick change mutagenesis on pRR51-gldL^E49A-E59A^)

5-BamHI-LM (for restriction) GCTAGGGATCCggaggcggaagaaaataataataacttagt

3-XbaI-LM (for restriction) GCTAGTCTAGAcgtggtatcattataagatattgaacgtag

5-GldM-E31A (for megapriming) CGCAATGTTAGCAATGAACGTATCAAAAgcaGTTATTTCTGCTTTTGGTTTGATGAATG

3-GldM-E31A (for megapriming) CATTCATCAAACCAAAAGCAGAAATAACTGCTTTTGATACGTTCATTGCTAACATTGCG

oTM495 CAACAATTAATAACTAATTATTatgaaaccagtaacgttatac

oTM496 GTACCTGCATGCtcactgcccgctttccagtc

oTM593 GAGATGCTAGCTTATTTGTATAGTTCATCCATGCC

oTM595 ATGTCTAAAGGTGAAGAACTG

oTM596 CAGTTCTTCACCTTTAGACATggttgctacGTTTTCACCAGT

oTM612 ttAATTggatccAACTAAATTGTAATTAAAAAAAATAAGTatgGGCTCTGGTGATGCATC

^a^ restriction site underlined.

^b^ mutagenized codons in red lower case.
