# Supplementary material for: Dynamic proton-dependent motors power type IX secretion and gliding motility in Flavobacterium
Source: PLoS Biol. 2022 Mar 25;20(3):e3001443. doi: 10.1371/journal.pbio.3001443 (PMC8986121; doi:10.1371/journal.pbio.3001443)
Supplement: Attachment — Submitted filename: Response to reviewers.pdf [file pbio.3001443.s007.pdf]

Itemized responses to Reviewer's comments

Dynamic proton-dependent motors power Type IX secretion and gliding adhesin movement in *Flavobacterium*

MS Vincent, C. Comas Hervada, C. Sebban-Kreuzer, H. Le Guenno, M. Chabalier, A. Kosta, F. Guerlesquin, T. Mignot, M. McBride, E. Cascales, T. Doan

(PBIOLOGY-D-21-02639)

We thank the reviewers for their very positive appraisal of our work and for their remarks and suggestions to improve our manuscript. We have performed the recommended experiments and have added this novel information and modified the text according to their comments.

The changes made are visible in the Tracked Changes document.

Reviewer #1

The manuscript by Vincent and co-authors builds on recent exciting developments understanding the structure and function of the T9SS motor in protein secretion and gliding motility within the Bacteroidetes phylum. Specifically this paper focuses on how PMF is converted by the GldLM inner membrane motor proteins into mechanical torque to the outer membrane apparatuses for secretion/motility. New findings include a GldL E59A substitution mutant that uncouples protein secretion from motility; NMR evidence for protonation of GldL glutamate residues within an IM channel involved in proton flux; uses genetic assay to map how TMHs in GldL & M interact; found dissipating the pH gradient with nigericin hinders motility; provided evidence that PMF influences GldM conformational state and that GldLM exist in stationary and mobile complexes in vivo. A number of experiments also confirmed prior results from the homologous P. gingivalis T9SS conducted by this group or results from other groups in related/identical systems. Overall the data fits well and builds upon other findings, including a recent report a by Hennell James et al, 2021, which reports on the structure and function of the GldLM proton-driven motor. In general, the experiments are clever, carefully done and the text is clearly written.

We thank the reviewer for the kind words and for the positive feedback.

Specific comments:
Fig. S2B. The TMH region shown for GldM is different than the one demonstrated in Hennell James et al, 2021 (see Fig. 2d). Importantly, E31 in Hennell James et al resides outside of the TMH and may explain why the E31A mutant had no phenotype. This discrepancy needs to be addressed/justified.
Indeed, based on the structural study by Hennell James and colleagues, the E31 residue in GldM resides outside of the membrane. This likely explains why the E31A mutation had no effect.

However, alternatively, we reasoned that PMF-dependent conformational shifts in GldM might bring E31 (which is perfectly conserved) within the membrane environment in a cyclic manner. To take the reviewer's comment into account, we have modified Fig. S2, which now indicates the TMH regions as defined by Hennell James and colleagues, and modified the text accordingly:

"Sequence alignment showed that three acidic residues are conserved in the GldL and GldM N-terminal regions (Supplementary Fig. S2A and S2B): a glutamate at position 31 in the vicinity of GldM TMH (GldM-E31) and two glutamates in GldL, one located in TMH2 (E49; strictly conserved in all GldL homologs in the OrthoDB database), and one located between TMH2 and the cytoplasmic domain (E59) (Supplementary Fig. S2C)"

Fig. 6A. The average number of foci per cell for GldL is shown. Since similar immunofluorescent experiments were done with GldM, GldK and GldN, their average foci number per cell should also be shown.

We have modified Figure 6A to show the mean number of foci for each labeling.

In Fig. 6B experiments, is it possible to quantify the fraction of foci that are static vs mobile? Additionally, at later time points (e.g. 1 to 10 min) do stationary foci become mobile and vice versa? That is, do specific GldLM complexes permanently reside as 'static secretion machines' or 'motile motility motors'?

These interesting questions are difficult to answer in the current state of our method.

First, NbALFA-sfGFP may not bind to all GldL-alfa molecules in the cell. Moreover, it may differentially bind to stationary (presumably in secretion complexes) and mobile GldL-alfa molecules (presumably not in secretion complexes). Therefore, quantification could be biased. Second, to determine if GldLM complexes can convert from static in secretion complexes to mobile motors functioning in gliding, we would need to track individual GldL-alfa molecules with good temporal resolution for a relatively long time. However, the sfGFP marker used here is sensitive to photobleaching. This type of question can be approached using photoactivatable or photoconvertible labels which are resistant to bleaching. We hope that the use of different labels in future experiments will provide further information.

Please provide and explanation for why NBalfa-sfGFP labeling of GldL-alpha produced a functional reporter, while in contrast various GldL and GldM fluorescent protein fusions did not. In the former case a much larger reporter complex is made; therefore it is not obvious why the latter constructs were non-functional.

The size of sfGFP is substantial (28 kDa, 2.4 nm in diameter). sfGFP steric hindrance may prevent the formation of GldL/GldM complexes or even the formation of higher order T9SS complexes. In addition, even if these complexes form, the sfGFP domain may perturb the function of the GldLM motors. By contrast, the alfa tag only adds 16 amino acid residues to GldL and is functional. One may hypothesize that NbALFA-sfGFP molecules bind to GldL-alfa which are already properly inserted, properly assembled into functional complexes.

The results with GldL-alpha/NbAlfa-sfGFP are intriguing but some controls are absent. (i) For example, a western of GldL-alpha to test if it is processed, which may explain why some populations are mobile, while the majority are static. (ii) NbAlfa-sfGFP reporter is a noncovalent interaction with GldL-alpha and therefore dissociates. Again, this could explain

why some foci are mobile. Given this is an atypical reporter, are there control proteins where the alpha peptide is fused to proteins that are known to be static and mobile? (iii) GldL-alpha is complexed with GldM, whose four extended domains span through the periplasm and cell wall. Therefore, there is an apparent conceptual issue for how GldLM could be mobile when it is predicted to be trapped within a peptidoglycan network.
The reviewer raises important points about our reporter. Below we provide additional information to address these issues.

-We have now included a western blot (new Fig. S3C) to show GldL-alfa production compared to wild-type GldL, probed with anti-GldL polyclonal antibodies or anti-alfa nanobodies. GldL-alfa does not appear to be processed in our conditions.

-The affinity of NBalfa for the alfa tag has been shown to be in the low picomolar range (Götzke et al, 2019 Nat Commun), indicating that dissociation rate is very slow. However, a certain number of NBalfa-sfGFP always remain unbound in the cell, inevitably generating a background signal. Since free NBalfa-sfGFP molecules are expected to diffuse very fast in the cytoplasm (in the order of 1-5 $\mu m^2/s$, Kapanidis et al 2018 J Mol Biol, PMID: 29753778), it is clearly not possible for us to detect discrete foci corresponding to free NBalfa-sfGFP molecules on our microscope and the "relatively long" exposure time used (100 ms). Hence, as shown in Fig. S3, free NBalfa-sfGFP produced in a cell without any alfa tag target generates a diffuse fluorescent signal at 100 ms exposure time.

- As the reviewer suggested, we tried to fuse alfa-tag to control proteins with predictable localization. This is actually tricky because we know very little about the localization of proteins in *F. johnsoniae* and candidate proteins are often essential. For example, we failed to generate fully functional fusions to the FtsZ (division septum) or MreB (cell wall elongasome) cytoskeletal proteins. In addition, we generated a functional GldM-alfa fusion. However, in vivo, NBalfa-sfGFP sent in the periplasm never bound to it. The tag may be hidden by GldM partner proteins.

-Fourth, to unambiguously attribute the fluorescence signal to the presence of GldL, we have evolved our reporter system towards a bimolecular fluorescence complementation approach using the recently described split-halotag (Minner-Meinen. Et al 2021 Plant Commun, PMID 34746759; Shao et al 2021 Communications Biology, PMID: 33742089). As shown below, GldL is now fused to the alfa tag and the short C-terminal domain (260-297) of the halotag self-labeling enzyme. This GldL-alfa-CterHalo fusion is functional. Then, NBalfa is fused to the large N-terminal domain (1-259) of halotag. In this construct, a functional halotag enzyme is reconstituted when and only when NBalfa-NterHalo binds to GldL-alfa-CterHalo, and can exhibit fluorescence in the presence of its fluorescent TMR ligand. As shown in the time lapse microscopy and kymograph below, we detected mobile and static fluorescent foci, indicating that there are static (red arrow) and mobile GldL-alfa-CterHalo molecules in the cell, similarly to what was observed with Gld-alfa detected with NBalfa-sfGFP in Fig. 6. As controls, NBalfa-NterHalo alone or GldL-alfa-CterHalo alone did not yield any fluorescence signal.
This split-halotag version is still preliminary and its fluorescence properties are poor and unstable compared to typical halotag labeling, requiring more work to be presented.

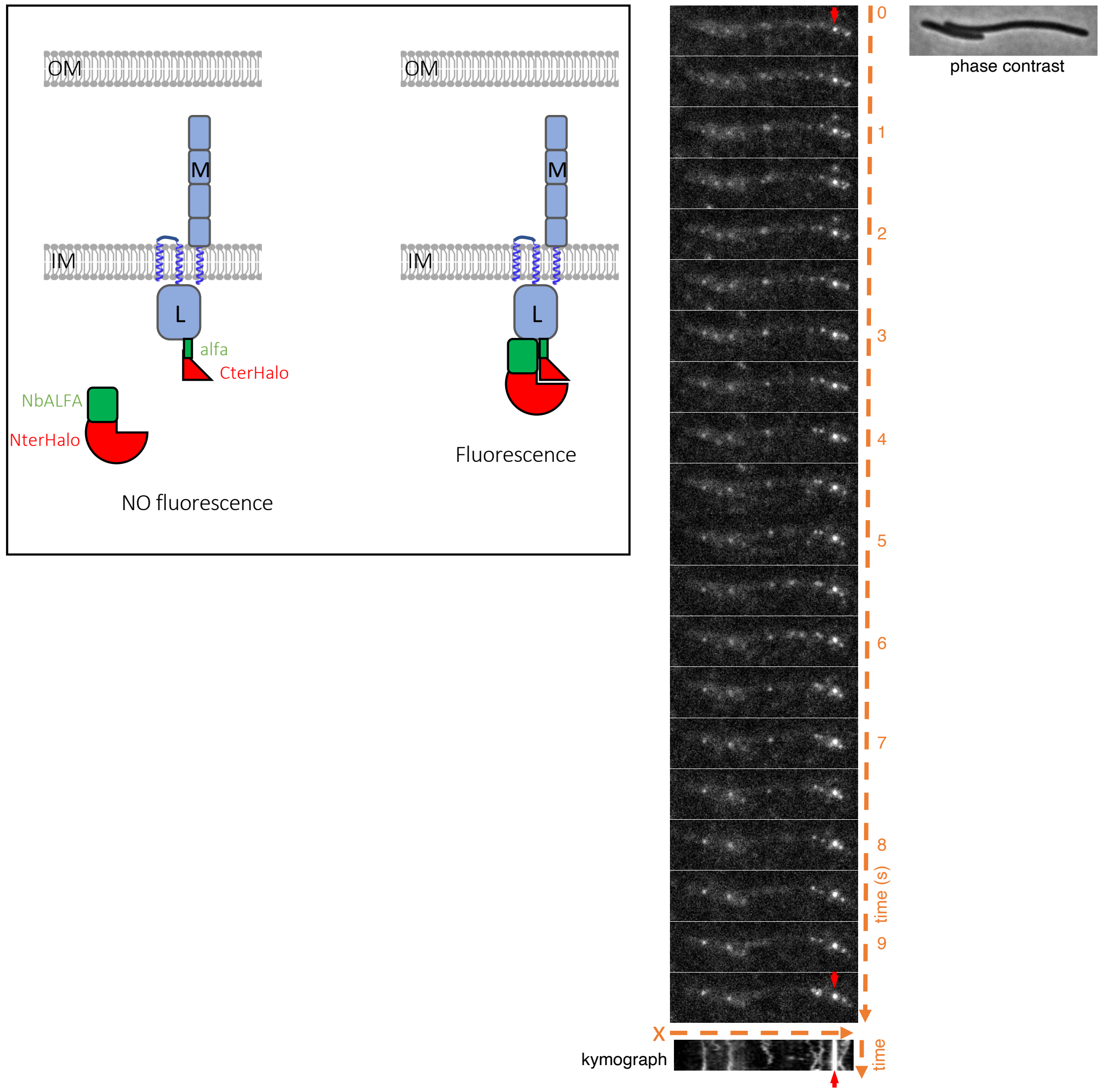

-Finally, the mobility of complexes that span the periplasmic space and therefore have to deal with the presence of the peptidoglycan mesh is a puzzling question. It applies here but also for other complexes such as the gliding apparatus of *Myxococcus xanthus*. It is known that the assembly of type III and Type IV secretion systems require specialized lytic transglycosylases to locally degrade the peptidoglycan meshwork in Gram negative bacteria. Therefore, one hypothesis is that lytic enzymes associate with GldLM motors and help displacement through the peptidoglycan.

Proteins/complexes in Fig. 6F/legend are poorly labeled/described. For example, is the gray IM cylinder the Sec machinery? If so, please label. Second, in the red OM cylinder, SprA is not described, but should be present as it serves as the channel for protein secretion. Third, what are the red/gray ovals under the red OM cylinder? Fourth, the secretion of SprB (red) is confusing. In the periplasm, it appears folded, while outside the cell it appears unfolded. Finally, what is the black triangle in the LPS layer (top), and what is the green hexagon in the OM and the green hatch below it in the bottom/right cartoon?
We thank the reviewer for this remark. Fig. 6F has been improved based on the comments above. We hope that the new model in Figure 6F fits better with the standards of the reviewer.

The significance of a sub-population of GldL (or GldLM) moving is unclear. To support or describe the "rack and pinion" model the GldLM motors could simply reside in fixed locations whereby they rotate/spin and hence move the "tread/track" that's presumably attached to the SprB cell surface adhesin.
A major weakness in the proposed "rack and pinion" model proposed by other authors and supported by these authors, is the lack of a known tread/track network required to translocate SprB on the cell surface.
The reviewer is correct. In the “rack and pinion” model, GldLM motors energize a track or a structure along the track to displace SprB. GldLM motors could be all fixed along the track, in which case the energy has to be transduced over relatively long distances, ie between GldLM fixed motors and SprB adhesins. Alternatively, GldLM motors could travel between different positions on the track, thereby reducing the distance or the number of motors required to move SprB along the entire track. In any case, we agree with this reviewer that the identification of a putative tread/track structure remains a big question in the field.

Lines 722-23: The Song et al reference lacks a journal name and lacks an actual citation in Google Scholar. Importantly, this reference includes authors from this group and appears to provide the first description/evidence for conformational changes in GldM, which is expanded upon in this work.
This citation refers to a manuscript describing the structure of entire T9SS complexes obtained by cryo-electron tomography. The manuscript has been submitted to another journal and is currently under revision. We would like to keep this reference in the text as it will take its full meaning soon (different conformations of GldM/PorM are visible in tomograms, depending of the energization status).

Movie S1 not provided.
Movie S1 was uploaded during the submission process. We will add a comment about this issue to the editor.

Minor comments:
The OM-associated ring is described two ways. On lines 81-82 it consists of GldK and GldN, while lines 171 & 882-3 it includes GldO. Please clarify.
In lines 81-82, we described the T9SS from *Porphyromonas gingivalis*. In this bacterium, the ring consists of homologs of GldK and GldN which were originally named PorK and PorN. For simplicity, we tried to use the “Gld” nomenclature throughout the manuscript. In lines 171 and 882-3, we specifically refer to the *Flavobacterium johnsoniae* ring. In addition to GldK and GldN, *F. johnsoniae* possesses GldO, a paralog of GldN. Hence, we refer to a GldKNO ring in *F. johnsoniae*, although it has not been formally shown in *F. johnsoniae*.

Lines 168-69: to aid the reader, please note here that the soluble domains of GldL and GldM reside in the cytoplasm and periplasm, respectively, so the finding that these domains do not interact is expected.
We modified the text as suggested by the reviewer.

Line 169: Please provide reference for the PorKLMN complex discussion.
The reference has been added.

Line 302: Please describe where in the alfa-tag was introduced in-frame in GldL in the Result/Discussion section.
We modified the text to be more precise:
"The sequence encoding the alfatag was introduced in frame at the C-terminus of the GldL-coding sequence at the native locus."

Lines 738-739. Kellenberger et al reference is listed twice (one of which is partial). Additionally, this reference is not cited in the text.
We corrected this reference issue.

In Fig. 2B, to aid the reader, label the N and C ends of GldL/M.
Done

Fig. 2C legend, line 786. For clarity after, "Measurements" insert "(transcriptional repression)." Also, lines 788-89, for clarity, after "Interactions with TssL1 (in gray) served as negative controls" add "or positive control for self-interactions."
Corrected

Fig. 6A, right graph. Change the y-axis description to "Number of foci/cell" if that is the intent.
Done

In Figs. 6B-E and S3B please state how long NBalpha-sfGFP was induced with IPTG prior to imaging.
The information has been added in the legend to figures.

Fig. 6D legend, line 870: What are "filled spots" and "empty spots?" I see blue and black circles (spots) in the line graph, but no empty spots.
The size of the spots has been slightly increased to better see the empty spots.

Reviewer #2

This is an interesting paper that describes the driving mechanism of the gliding motility and the type 9 secretion of Flavobacterium johnsoniae.

The gliding motility and the type 9 secretion are powered by PMF. In this study, the authors clearly indicated that gliding motility is powered by the delta pH component of PMF but not

by the membrane potential component. An inner membrane complex, GldLM, is believed to be a proton channel and act as a motor to drive these processes. The authors demonstrated that glutamate residues (E49 and E59) in the transmembrane helix 2 of GldL are important for the GldLM function. Interestingly, E59 is essential for the gliding motility but not for type 9 secretion, whereas E49 is essential for type 9 secretion. Moreover, they revealed localization and movement of GldLM and SprB, and their data support the tread model. These findings greatly contribute to our understanding of the mechanism of the gliding motility as well as the type 9 secretion.

We thank this reviewer for the very positive appraisal of our work.

However, I found some issues that should be clarified before publication.

(1) The result of limited proteolysis shown in Fig.3A seems inconsistent with that in Fig. 4H. Addition of CCCP (proton ionophore) changed the GldM structure to a protease sensitive form (Fig. 3A). However, the gldL E59A mutation, which may block the proton channel for gliding motility, did not change GldM to the protease sensitive form (Fig. 4H).
The reviewer raises a very interesting point. In the GldL E59A mutant, the motor is functional for T9SS-dependent secretion, as shown with the SprB substrate (Fig. 4F). Hence, in this mutant, the proton channel functions at least partially, thereby the GldL E59A proteolysis profile is similar to the wild-type profile. This suggests that in response to the PMF, E59 in GldL might serves an additional conformational shift only required for gliding.

(2) Line 198-200
This is not true. A kink between PorM D1 and D2 has been found in the crystal structure of the entire periplasmic region of PorM. "Hennell James, Deme et al" observed PorLM and did not show the D1-D2 kink of GldM in their CryoEM structure.
The reviewer is totally correct. We apologize for this shortcoming. Hennell James did not show the D1-D2 kink in GldM. They proposed a composite model in which the bend between D1 and D2 "would allow the tip of a rotating GldM dimer to define a large track at the outer membrane". The text has been modified accordingly.

(3) Fig. 3B
The direction of the black arrow may be wrong.
We totally agree. The arrow direction has been switched!

(4) Line 254-
Did peptides form a complex like the GldL5+GldM2 complex in the micelles? If not, I think it is nonsense to discuss the protonation of the glutamate.
The NMR approach we performed shows that GldL and GldM TMHs interact in DPC micelles using a L1:L2:M ratio of 1:1:1. With such a ratio, it is unlikely that these interactions respect the stoichiometry observed by Hennell James *et al.* in the fully-assembled GldLM complex. However, what we mainly point out is the influence of the protonation state of GldL glutamates on the interactions between TMHs, which can be directly assessed by NMR.

(5) Line 722 "Song, Perpich et al. 2021".
Incomplete description. I could not find the reference paper.
As answered to reviewer 1, this citation refers to a manuscript describing the structure of entire T9SS complexes obtained by cryo-electron tomography. The manuscript has been submitted to another journal and is currently under revision. We would like to keep this reference in the text as it will take its full meaning soon (different conformations of GldM/PorM are visible in tomograms, depending of the energization status).

(6) Fig.1
How much time do you needed to recover the motility after washing CCCP out?
In our hands, recovery of motility was almost immediate, within 10 s. A similar observation was reported earlier by the group of Koji Nakayama (Nakane et al, 2013 PNAS). They observed that the cells stopped moving after 3 s after addition of CCCP and resumed gliding within 6 s after removal of CCCP. This suggests that the gliding apparatus is fully preserved in the presence of CCCP but the “fuel” is not available for the GldLM motors.
